# Distinguishing Discoid and Centripetal Levallois methods through machine learning

**DOI:** 10.1371/journal.pone.0244288

**Published:** 2020-12-23

**Authors:** Irene González-Molina, Blanca Jiménez-García, José-Manuel Maíllo-Fernández, Enrique Baquedano, Manuel Domínguez-Rodrigo

**Affiliations:** 1 IDEA, Institute of Evolution in Africa, Universidad de Alcalá de Henares, Madrid, Spain; 2 Artificial Intelligence Department, Universidad Nacional de Educación a Distancia, UNED, Madrid, Spain; 3 Department of Prehistory and Archaeology, Universidad Nacional de Educación a Distancia, UNED, Madrid, Spain; 4 Museo Arqueológico Regional, Alcalá de Henares, Madrid, Spain; Ghent University, BELGIUM

## Abstract

In this paper, we apply Machine Learning (ML) algorithms to study the differences between Discoid and Centripetal Levallois methods. For this purpose, we have used experimentally knapped flint flakes, measuring several parameters that have been analyzed by seven ML algorithms. From these analyses, it has been possible to demonstrate the existence of statistically significant differences between Discoid products and Centripetal Levallois products, thus contributing with new data and a new method to this traditional debate. The new approach enabled differentiating the blanks created by both knapping methods with an accuracy >80% using only ten typometric variables. The most relevant variables were maximum length, width to the 25%, 50% and 75% of the flake length, external and internal platform angles, maximum width and number of dorsal scars. This study also demonstrates the advantages of the application of multivariate ML methods to lithic studies.

## Introduction

Identifying specific blanks of débitage and flaking methods is frequently a complex task. This is because blanks can be obtained by several completely different methods, as exemplified by Levallois points [1], or because the flakes or cores from different methods converge in shape and size and can be difficult to differentiate. Several studies have aimed to morphologically and qualitatively identify some of these methods through the technological, diacritical, and structural readings of blanks and cores [2–4], and, more recently, through various methodologies such as typometry, statistics, 3D models, and geometric-morphometric approaches [5–14].

One of these examples is the debate about the blanks obtained by Discoid and Centripetal Recurrent Levallois methods (Centripetal Levallois). Several researchers have remarked the difficulty in discerning between the flakes and cores of both methods as they are similar in many of their characteristics [3, 15–18]. This has led some researchers to consider it appropriate to unify both methods within the same name "Recurrent Centripetal flaking method" [19].

The importance of discerning between both methods is not a minor issue since, although we consider both as prepared core methods, the techno-cultural value that is given to Levallois methods to define the Middle Palaeolithic and the Middle Stone Age [20, 21] is paramount.

In this work we address the debate on the Discoid and Centripetal Levallois concepts, proposing a new way of identification of both methods by using a specific set of variables and classification ML algorithms tested against experimentally controlled collections. ML has provided an increase in the efficiency of handling multivariation in archaeological information. In the past five years, there has been a blooming of ML techniques in taphonomic research [22–25] as well as in paleontology [26, 27]. The use of ML has significantly increased the accuracy in classification problems in those fields. The plasticity of ML algorithms and their diversity makes them excellent candidates for classification problems. Here, we will use some of the most relevant algorithms to identify their heuristics when handling multivariate information stemming from controlled experimentally-flaked collections created for the problem defined below.

## Levallois and Discoid concepts

### Levallois

Levallois methods were defined by V. Commont from his works on the Late Acheulean in the Somme Valley [28–30], although it was the works of F. Bordes from 1950 onwards that marked the beginning of the modern studies of these methods [31–33]. Bordes defined Levallois flakes as flakes that were predetermined by a special core preparation, with a striking platform that could be plain, dihedral or faceted. Levallois cores would be prepared from extractions in a centripetal direction, usually on flint nodules whose initial shape should be oval or flattened, leaving a shape that would resemble a “tortoise core.” At one of the ends of the core would be the striking surface, which is prepared so that it remains more or less perpendicular to the axis of the core which serves as an intersection between the two surfaces. The knapping action at this point would generate an oval-shape flake with centripetal scars on its dorsal surface [31, 33]. The intentionality in obtaining a predetermined flake was for Bordes the essential criterion that would distinguish the Levallois débitage from other flaking methods [34].

Bordes' definition laid the groundwork for posterior studies. However, those were not without limitations, which were soon exposed, namely by the subjectivity in their interpretation and because they relied on the researchers’ experience [5, 35].

From a technological point of view, Eric Boëda would redefine the Levallois method by establishing six predetermination criteria [1]: (a) the core’s volume is conceived as two convex asymmetric secant surfaces, whose intersection defines a plane; (b) the surfaces are hierarchical: one functions as a débitage surface and the other one as a striking surface; (c) the débitage or exploitation surface has lateral and distal convexities that must be maintained; (d) the fracture plane of the predetermined flakes is parallel to the intersection between both surfaces; (e) the striking surface is oriented so that the intersection between both surfaces is perpendicular to the length axis; (f) the technique is executed by direct percussion using a hard hammer.

We agree with some colleagues who consider these criteria as a “theorical optimum” that can be undermined by numerous variables such as raw material, knapping accidents, skills of knappers, among others [10].

Therefore, in the Levallois conception there are two groups of methods: preferential and recurrent. In the former, a flake is produced per each prepared surface. This includes the “Preferential”, the “Points” and the “Nubian” methods [36, 37]. The latter produces several Levallois blanks per prepared surface, including the parallel Unidirectional, Convergent Unidirectional, Bidirectional Parallel and Centripetal methods [1]. It is the latter, the Centripetal Levallois, that has generated the most discussion regarding its relationship with the Discoid method.

The blanks created by Centripetal Levallois are varied both morphologically and metrically. The panoply of forms varies from symmetric blanks in the first series of débitage (similar to the preferential Levallois flakes), to atypical, centripetal, *débordant* Levallois flakes (with cortical backing or not) and pseudolevallois points [38].

### Discoid

Discoid methods are undoubtedly the most common débitage conception within prepared cores. Again, F. Bordes was the one who first defined it, calling it Mousterian débitage [31, 33]. He emphasized its similarity to the Levallois cores but pointed out the possible bifacial exploitation [33]. It could be argued that the true definition came in the 1980s with the studies of Guilbaud [39] and Gouëdo [40], among others.

In the mid-1990s, E. Boëda defined the Discoid conception, differentiating it from the Levallois, but only recognizing bifacial methods as discoid and, although he did not consider it as a predetermined débitage method at first [1, 41], he does so today [4]. Currently, many researchers consider this method as a premeditated conception of débitage, such as the Levallois methods, given that the premises of the volumetric concept of exploitation and selection of raw materials are similar [17, 18, 42]. On the other hand, more Discoid methods have been documented than the strictly bifacial, such as the unifacial (with hierarchical surfaces) or multifacial [17] and which formed the so-called Discoid *sensu lato* [43].

The Discoid conception is defined based on a series of criteria [17, 41]: (a) the volume of the core is conceived in two asymmetric, secant and convex surfaces that delimit an intersection plane; (b) both surfaces may or may not be hierarchical, (c) the exploitation or débitage surface is designed with a peripheral convexity that controls the knapping of each extraction, (d) the fracture plane of the predetermined flakes is secant at the intersection between both surfaces (e) the striking surface is oriented so that the intersection between both surfaces is perpendicular to the edge of the core, (f) the technique is direct percussion using a hard hammer.

**Fig 1:**
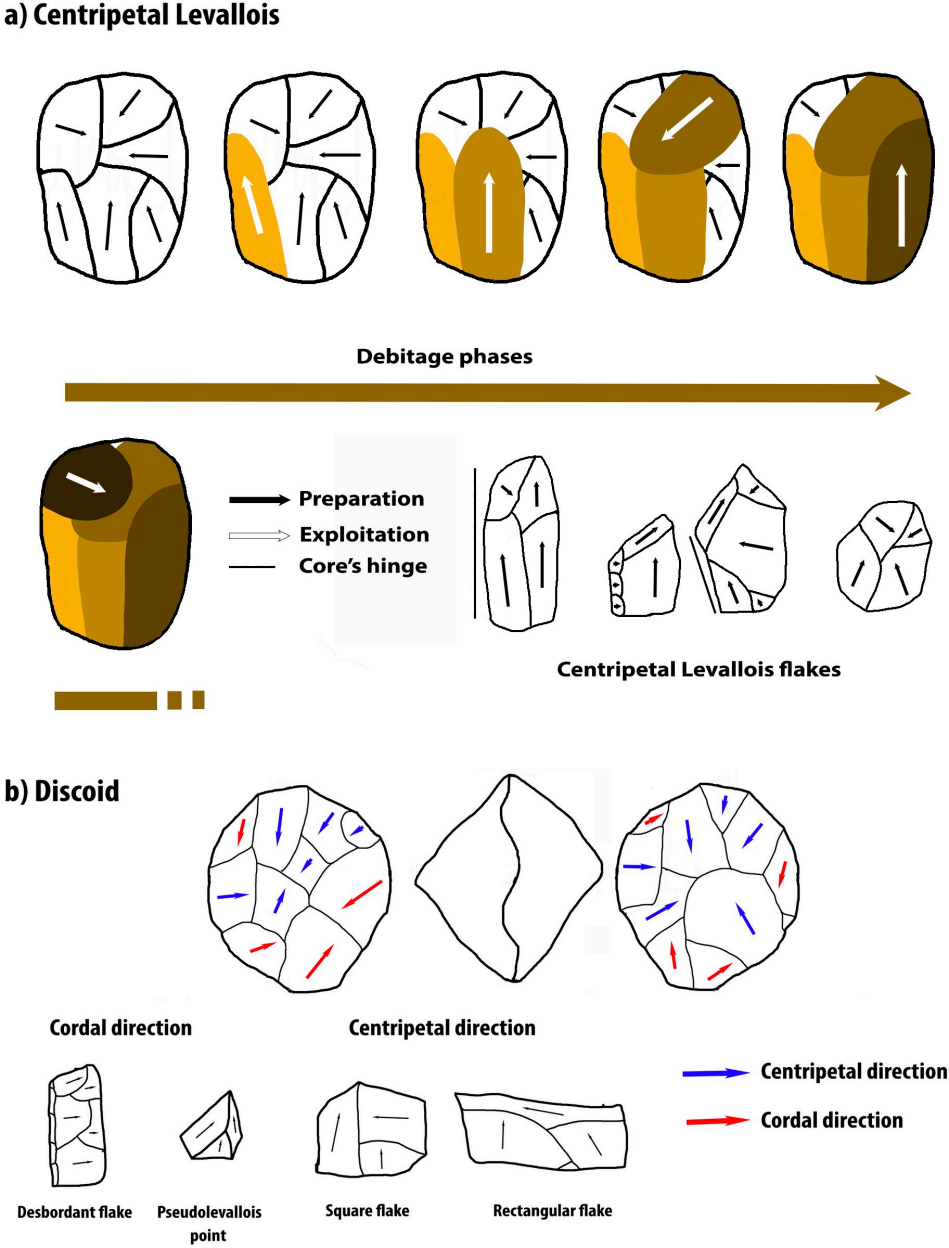
Centripetal and Discoid débitage rhythm, directions and main blanks types. A) Modified from [37].

The débitage can have two directions in the Discoid conception: cordal (the axis does not pass through the center of the core) or centripetal (the axis passes through the center of the core). By combining these two directions, proper convexity can be maintained for uninterrupted flake production, as the cordal flakes create convexity on the débitage surface and the centripetal flakes destroy it. Thus, we could speak of a "self-maintenance" and it is not necessary to stop to redesign the predetermination criteria as it happens with the Levallois methods, except for the Levallois Centripetal. The blanks obtained would thus be pseudo-levallois points and *débordant* flakes, which would have a cordal direction; and wider than long and quadrangular flakes, of centripetal direction [41 and Fig 1].

### Centripetal Levallois *versus* Discoid

The comparative analysis of the Levallois and Discoid conceptions is extensive in aspects such as productivity and efficacy of their blanks [10, 44–48]. However, we are going to focus on the aspects that are related to the objectives of our analysis: morphological similarity, characteristic parameters of the concept and degree of predetermination.

To solve this, Boëda proposed the methodological criteria that define the predetermination of the Discoid and Levallois concepts that we have pointed out before. However, these criteria were soon questioned for various reasons [17, 18, 49] related to discoid criteria in relation to the Levallois ones [1, 41] and which are summarized in Table 1.

**Table 1:** Predetermination criteria for Discoid and Levallois Methods Centripetal Recurrent. From [18, 41].

	DISCOID	CENTRIPETAL LEVALLOIS
**a**	the volume of the core is organized around two symmetrical or asymmetrical convex and drying surfaces	Idem
**b**	both surfaces may or may not be hierarchical	the two surfaces are hierarchically related
**c**	The debitage surface has a peripherical convexity.	The debitage surface has lateral and distal convexities.
**d**	The orientation of stricking platform is paralelle to line between both surfaces	Idem
**e**	the fracture plane of the discoid flakes are secants to the plane of intersection of the core’s two surfaces	the fracture plane of the Levallois flakes is parallel to the plane of intersection of the core’s two surfaces;
**f**	the technique is direct percussion using a hard hammer	Idem

The secant plane between the two surfaces of the discoid cores has been questioned, since the existence of discoid cores whose fracture plane is parallel or subparallel to the intersection between the two surfaces has been reported, producing large and thick blanks [49] or that the surfaces are less secant as the thickness of the core decreases [50].

Regarding the convexity of the cores, Boëda points out as a difference between both conceptions that in the Centripetal Levallois, the convexity is lateral and distal, while in the Discoid method the convexity would be central [41]. However, Lenoir and Turq [19] point out that centripetal Levallois cores have a central convexity (like discoids). In turn, in the final stages of some discoid cores, a large invasive flake can be obtained, which is, morphologically, similar to some Levallois cores [18]. However, this scar occurs already outside the criteria of Discoid débitage [4].

Hierarchization, one of the differentiating elements of both concepts of débitage [1, 41], has also been questioned by some authors. Some Discoid methods are documented (among them the unifacial method with hierarchical surfaces) as Levallois [17, 18]. Thus, in many cases, unifacial discoid cores can be morphologically mistaken with Levallois cores [50, 51].

In addition to the characterization criteria already discussed, part of the debate between the two concepts of débitage has focused on the degree of predetermination, especially of the Discoid. There are many researchers who consider that the Discoid conception presents a weak predetermination producing poorly standardized blanks [19, 49, 52] and that these are obtained by activities to maintain the débitage chain [53].

We suggest that the debate on this point is poorly focused and should center on the definition of predetermination, which does not have to admit, *a priori*, that it is low or high. Predetermination is behind every technical human action, since behind every technical action there is a mental projection with more or less mental anticipation [15, 17]. Therefore, the Discoid conception, being recurrent, is already predetermined since the preparation of the core, although it is simple in its conception [4, 15]. Thus, admitting that similar blanks can be obtained from different methods, such as in Centripetal Levallois and Discoid, the differences between both methods lie in the architecture of the cores and in the preparation modes, even though morphologically they may be similar when abandoned. Thus, we must assume that the blanks obtained from both methods will have these conceptual differences, although they are morphologically similar and difficult to discern, as many researchers have shown [5, 19, 35].

Finally, different authors see a difference between both methods regarding the débitage chain: continuous for the Discoid and discontinuous for the Levallois due to the clear difference between the preparation and exploitation phases [54]. This argument has been refuted for Centripetal Levallois, where the maintenance of convexities through *débordant* flakes makes it possible not to interrupt the débitage process [55].

## Methods and materials

The aim of this article is to bring a new perspective to the Discoid-Levallois debate, through the application of ML algorithms to discriminatory variables elaborated on a controlled experimental collection. We believe that these algorithms, which have already demonstrated their effectiveness in other fields of archaeology (such as spatial archaeology or taphonomy), can also be very useful in the study of lithic industry thanks to their high capacity for classification, superior to other methods [22–27]. For this purpose, we assume that the Discoid and the Centripetal Recurrent Levallois are two different methods, with different volumetric conceptions, despite the fact that sometimes they may be similar in their morphology. Therefore, under this assumption, in the products obtained through both methods there should be qualitative and quantitative differences that should be statistically discriminated.

### Materials

In order to make a controlled experimental collection for this study, we knapped a total of six cores in different types of flint, three of them using the bifacial discoid method and the other three using the Centripetal Levallois method. All of them knapped by a single expert flintknapper. For this experiment we used fine-grained flint from different sources, with oval morphology and with cortex. We used 3 hammers: two small quartzite hammers (<10 cm long) with 262 grams (g) and 373 g of weight and one in sandstone (112 g) that was used for abrasion. The knapping sequence was typical of the prepared cores: a core preparation phase (more intense in the Levallois methods) and an exploitation phase. The technique used was exclusively direct percussion with a hard hammer. Flakes with knapping accidents were studied too. Only complete flakes from exploitation phase were considered. A total of 107 flakes were analysed (Table 2 and S1 Text).

**Table 2:** Discoid/Centripetal Levallois’ experimental lithic inventory.

Method	Core	Flint type	Nodule weight (g)	Flakes	Exploitation Flakes weight (g)	Total
**Discoid**	1	Flysh (Spain)	683	19	216	61
	2	Flysh (Spain)	108	13	51	
	3	Bergerac (France)	1001	29	673	
**Centripetal Levallois**	1	Molina de Aragón (Spain)	976	19	320	46
	2	Norwich area (UK)	519	7	78	
	3	Madrid area (Spain)	1775	20	1024	
**TOTAL**						107

**Fig 2:**
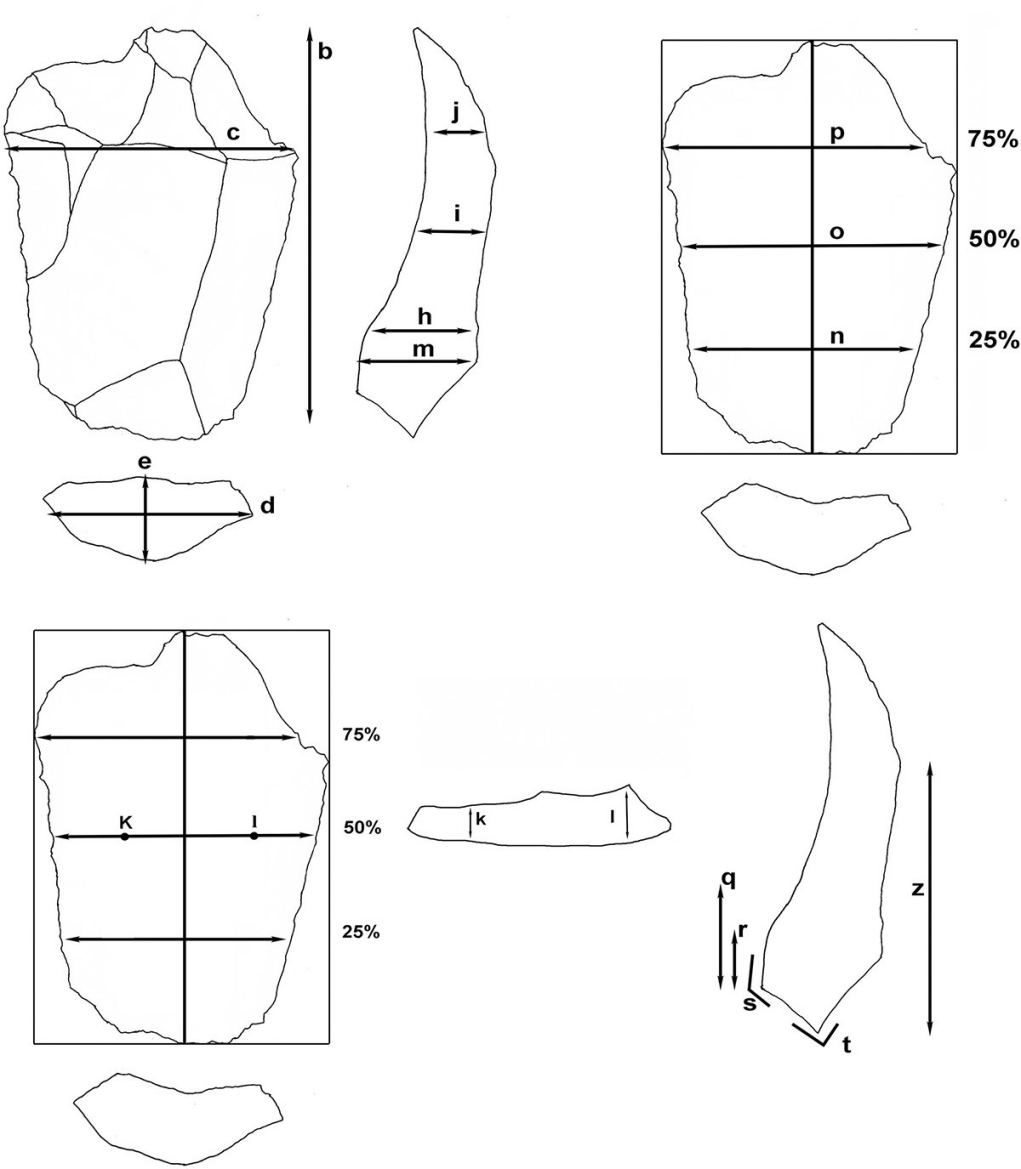
Flake variables measured for analyses.

The total number of attributes considered in this study is 28. The parameters applied are those usually used in standard typometric studies and the following are specific to this analysis [9] (Fig 2):

a) Cortex. On the dorsal surface of a flake: without cortex (0) or with cortex (1).

b) Maximum lenght (technological axis). Measured in millimeters (mm).

c) Maximum width. Measured in millimeters. The measurements were taken from the leftmost point to the rightmost point.

d) Platform lenght. Measured in mm.

e) Platform width. Measured in mm.

f) Platform type. We distinguish between plain (1), dihedral (2), facetted (3), indeterminate (4) or punctiform (5).

g) Lip. The lip absence (0) or presence (1) between the ventral surface and the platform.

h) Thickness at 25% of length. Measured in mm. At the midpoint of maximum width, we drew a perpendicular line representing maximum length. On this line, we measured the point where 25% of maximum length is located.

i) Thickness at 50% of length. Measured in mm, it matches with the 50% of width.

j) Thickness at 75% of length. Measured in mm.

k) Thickness at 25% of maximum width. Measured in mm. It is measured on a perpendicular line at 50% of lenght.

l) Thickness at 75% of maximum width. Measured in mm.

m) Maximum thickness. Measured in mm.

n) Width at 25% of length. Measured in mm.

o) Width at 50% of length. Measured in mm.

p) Width at 75% of length. Measured in mm.

q) Bulb length. Measured in mm. It is measured from the impact point to the point where the curvature of the bulb ends.

r) Distance from the bulb maximum point to the platform. Measured in mm, we measured the distance from the thickest point of the bulb to the platform.

s) Inner angle. Measured in degrees. The angle between the platform and the bulbar surface is measured.

t) External angle. Measured in degrees. The angle between the platform and the dorsal surface is measured.

u) Number of negatives.

v) Direction of negatives (previous scars). We distinguish between centripetal direction (1), distal (2), distal-lateral (3), proximal-lateral (4), proximal (5) and bidirectional (6). See diagram in S2 Text.

w) Cross section. Morphology of the cross section of the flake. We differentiate between trapezoidal (1), triangular (2), rectangular (3) and convex (4).

x) Morphology. Morphology of the flake as seen from its dorsal surface. We distinguish between trapezoidal (1), triangular (2), rectangular (3), semi-circular (4), rhomboidal (5), circular (6) and squared (7).

y) Index of symmetry [56]. The formula used is $S=\sum_{i=1}^{n}\left(\frac{\sqrt{{\left(Xi-Yi\right)}^{2}}}{Xi+Yi}\right)$, which eliminates the size factor. Xi is equivalent to the distance from the leftmost point to the imaginary line that represents the technological axis, and Yi from the rightmost. It is measured at 25%, 50% and 75% of length, solving the equation in each case and adding up the three results. The closer the result is to 0, the more symmetrical the flake is (Fig 3).

**Fig 3:**
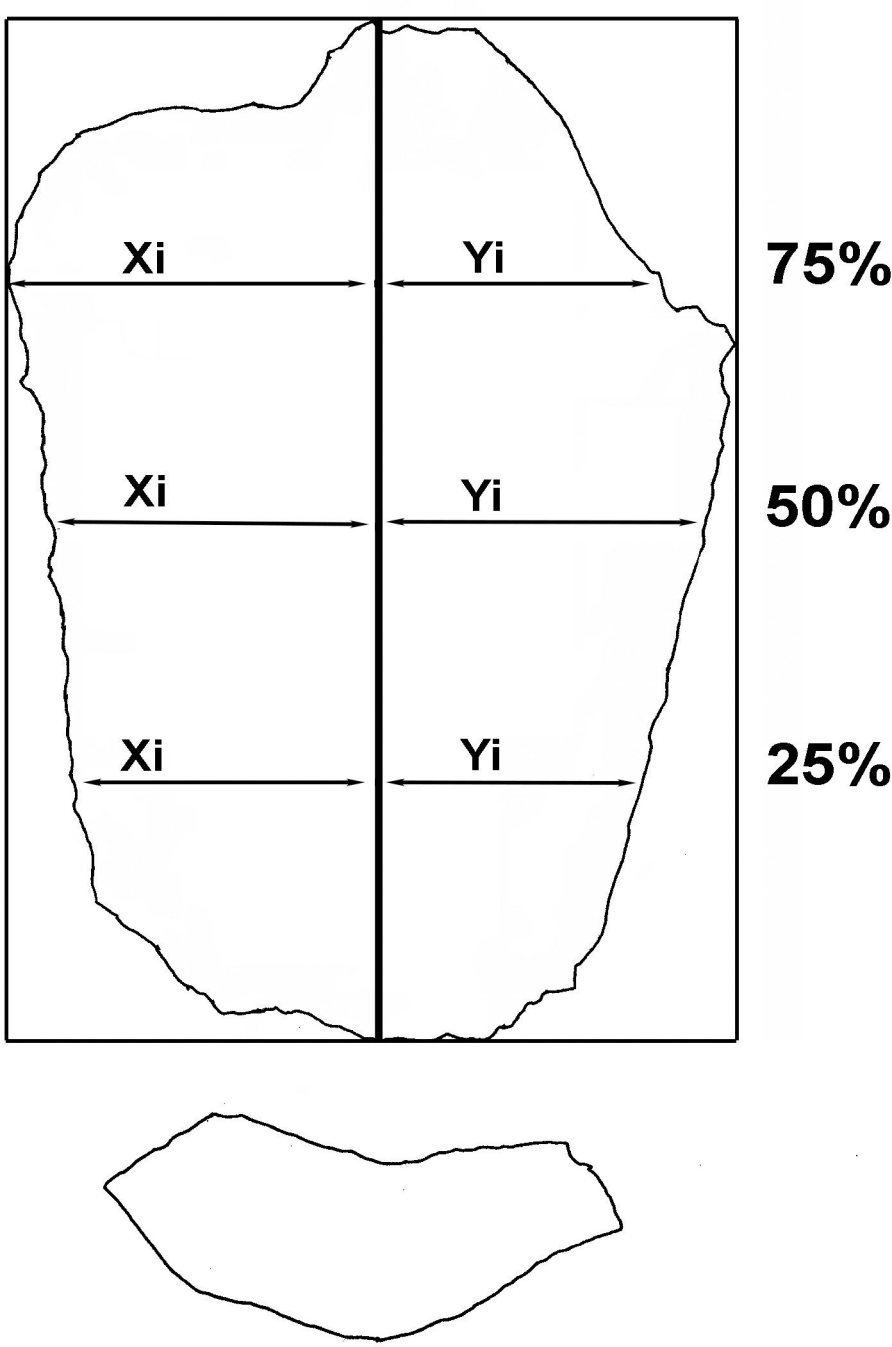
Flake variables measured for symmetry index.

z) Point where the negative of convexity begins. Some flakes end abruptly with a negative that marks the point where the flake takes away part of the convexity of the core. It is measured in mm from the platform to the point where this negative begins. This parameter was finally discarded.

aa) Débitage direction. We distinguish between cordal (1) or centripetal (2). Refits were made in order to know the exact place in the *chaîne opératoire* and the débitage direction of each flake.

ab) Technology classification. We distinguish between cordal flake (1), centripetal (2), pseudolevallois point (3), *débordant* (4) and Centripetal Levallois flake (5). See definitions in S3 Text.

### Methods

ML techniques have not been applied to archaeological questions until recently; however, they have shown their great usefulness and accuracy when dealing with classification problems that were challenging using more traditional methodologies [22–24, 57, 58].

Our study was developed in two stages: firstly, we compared seven different algorithms using R software version R-3.4.4 (www.r-project.org), being our null hypothesis that there are no remarkable differences between Discoid and Centripetal Recurrent Levallois methods. This first analysis was performed using 27 out of 28 measured variables, since the distal convexity attribute was discarded because of its absence in multiple pieces. Then, for the second stage, we carried out a hyperparameter optimization in the highest resolution algorithm in order to improve its performance.

### ML methods

#### Support Vector Machine (SVM)

It is a nonlinear classification method that can achieve a high accuracy in classification tasks. This method creates a hyperplane, a mathematical and spatial boundary that separates the data in a homogeneous space. Using kernels, additional dimensions are added to the data, so that a competent separation is achieved [22]. To implement this algorithm the “e1071” and “caret” R libraries were used.

#### K-nearest neighbor (KNN)

This supervised learning method classifies labeled data by assigning them to the most common class among its nearest neighbors. It performs especially well on samples with many variables and well-defined label sets. Different models are tested and an intermediate value is generally selected [59]. It requires the R “class” library.

#### Random Forest (RF)

It is an ensemble method that averages sets of decision trees, using a small randomly-selected set of variables. Through a bagging process focusing of different sets of discrete variables every time, a data set is bootstrapped to generate several complete trees that are afterwards tuned considering the average error in order to improve the performance; the final tree is the sum of all the previous ones. Although this technique requires more computation, it improves the result, not only because it averages several trees, but also because it avoids overfitting. It also uses the OOB (“out of the bag”) technique; this way, part of the sample not used for the training data set–or out of the bag observations–is withdrawn (33%) for a validation test, creating a natural cross-validation process and indicating the optimal number of iterations to minimize de OOB error. Importance of variables is stablished through the Gini index, which is the most adequate when dealing with categorical variables [57]. Here, the R “Random Forest” and “caret” libraries were used.

#### Mixture Discriminant Analysis (MDA)

It is an extension of linear discriminant analysis and it is based on class-specific distributions combined into a single Gausian distribution. This is done by creating a per-class mixture, as described by Kuhn and Johnson [60]. This consists of separating the class-specific means from the class-specific covariance structure. Otherwise described, each class has different means but the complete-class data set has the same covariance [22].

#### Naive Bayes (NB)

Bayes´ Rule, as used in the NB algorithm, estimates probabilities of classes on observed predictors (i.e., probabilities of previous outcomes), resulting in dynamic estimates of posterior probabilities of classes. Predicted classes are created based on the largest class probabilities for each class as derived from the training set. [23]. The R libraries "e1071" and "klaR" were used for this algorithm.

#### Partial least squares discriminant analysis (PLSDA)

This test classifies classes by identifying the predictor combinations that optimally separate classes. PLSDA finds latent variables (components) that maximize classification accuracy. Therefore, when data reduction is required for classification, PLSDA is preferred over PCA or LDA.[22]. For its implementation, the R "pls" and "caret” libraries are required.

#### Decision Tree using C5.0 algorithm (DTC5.0)

This method achieves an accuracy comparable to much more complex machine learning methods, such as neural networks or support vector machines. It works by recursively partitioning data. Performance can be improved with meta-learning methods, such as k-fold cross validation. We used the “C50” and “caret” R libraries for this analysis.

When comparing the different classifiers, we considered the most commonly used measures–accuracy, kappa value, 95% Confidence Interval, specificity and sensitivity, explained in detail in S4 Text–. A training set was created to evaluate the ML algorithms and a testing set was used to check their accuracy, specificity and sensitivity. A total of 70% of the original sample was used for the training model. Testing/validation was carried out on the 30% remaining sample. This is a standard procedure in predictive models in order to deal with the bias/variance tradeoff [23]. Each ML test carried out on the training set was also trained using cross-validation methods. This also contributes to balance bias and variance.

As explained in Domínguez-Rodrigo [23], each model cross evaluation takes place through the selection of subsamples of the original data and fitting them in multiple submodels. The results of these submodels is aggregated and averaged. Several techniques can be used for this subsampling and submodeling: generalized cross-validation, k-fold cross-validation, leave-one-out-cross validation or bootstrapping. Here we selected 10-fold cross validation, which consist of the original sample being partitioned into 10 similarly-sized sets. A first model is subsequently generated using all subsamples but the first fold. Then the first subset is reintroduced to the training set and the procedure is repeated with the second fold and so on until the tenth one. The estimates of performance of each of the ten processes are summarized and, thus, used to understand the model utility.

## Results

All the algorithms showed an accuracy in classification over the 80% of the testing set. RF outperformed the rest with a 90% accuracy in differentiating discoid flakes from centripetal recurrent Levallois flakes. DTC5.0 and MDA also showed remarkably high accuracy, correctly classifying 86.7% of the testing set. The least useful algorithm when discriminating between products of both methods was NB, even though it reached 80% of accuracy. As for KNN, SVM and PLSDA, all three correctly classified correctly 83% of the testing sample. The details about sensitivity and specificity, as well as the rest of parameters, of the models are shown in Table 3.

**Table 3:** Details on the performance of the seven Machine Learning algorithms compared.

Algorithm	Accuracy	Kappa	95%CI	Sensitivity	Specificity
Support vector machine	83%	0.65	0.65–0.94	0.89	0.75
K-nearest neighbor	83%	0.65	0.65–0.94	0.89	0.75
Random Forest	90%	0.79	0.73–0.98	0.89	0.92
Mixture discriminant analysis	87%	0.71	0.69–0.96	0.94	0.75
Naive Bayes	80%	0.59	0.61–0.92	0.78	0.83
Partial least squares discriminant analysis	83%	0.67	0.65–0.94	0.78	0.92
Decision tree with C5.0 algorithm	87%	0.72	0.69–0.96	0.89	0.83

In order to ease replicability, we carried out a second analysis attempting to reduce bias through hyperparameter optimization; this technique searches for the best combination of parameters of any given algorithm in order to optimize the validation loss and improve its performance [59]. As a result, SVM and MDA improved their performance and reached 93% accuracy in the classification, whereas RF and DTC5.0 achieved a 90% of accuracy, and KNN, NB and PLSDA stayed the same (Table 4).

**Table 4:** Details on the performance of the seven Machine Learning algorithms compared after hyperparameter optimization.

Algorithm	Accuracy	Kappa	95%CI	Sensitivity	Specificity
Support vector machine	93%	0.86	0.78–0.99	1	0.83
K-nearest neighbor	83%	0.65	0.65–0.94	0.89	0.75
Random Forest	90%	0.79	0.73–0.98	0.89	0.92
Mixture discriminant analysis	93%	0.86	0.78–0.99	1	0.83
Naive Bayes	80%	0.59	0.61–0.92	0.78	0.83
Partial least squares discriminant analysis	83%	0.67	0.65–0.94	0.78	0.92
Decision tree with C5.0 algorithm	90%	0.79	0.73–0.98	0.94	0.83

**Fig 4:**
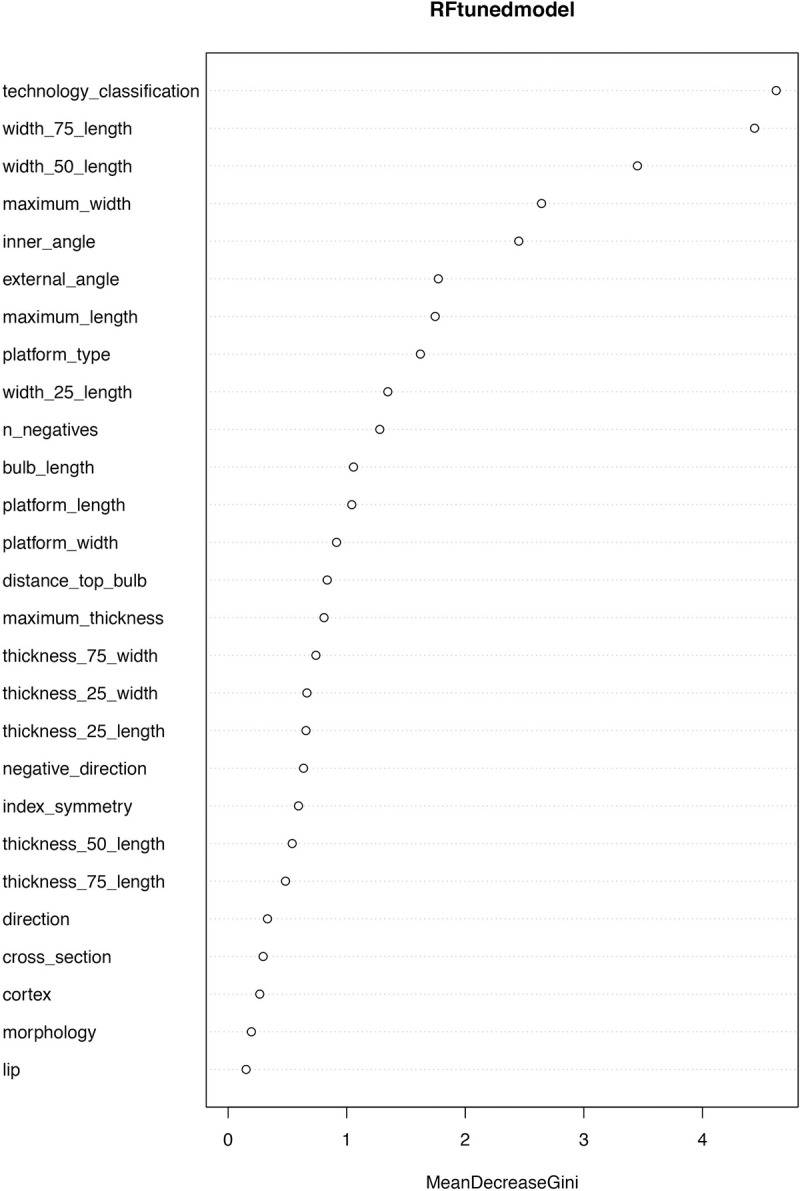
Importance of variables for the first tuned model of Random Forest. Here, “MeanDecreasGini” measures the total decrease in node impurity at each split, weighted by the proportion of samples reaching that node in each individual tree. The more the Gini Index decreases for a feature, the more important it is.

**Fig 5:**
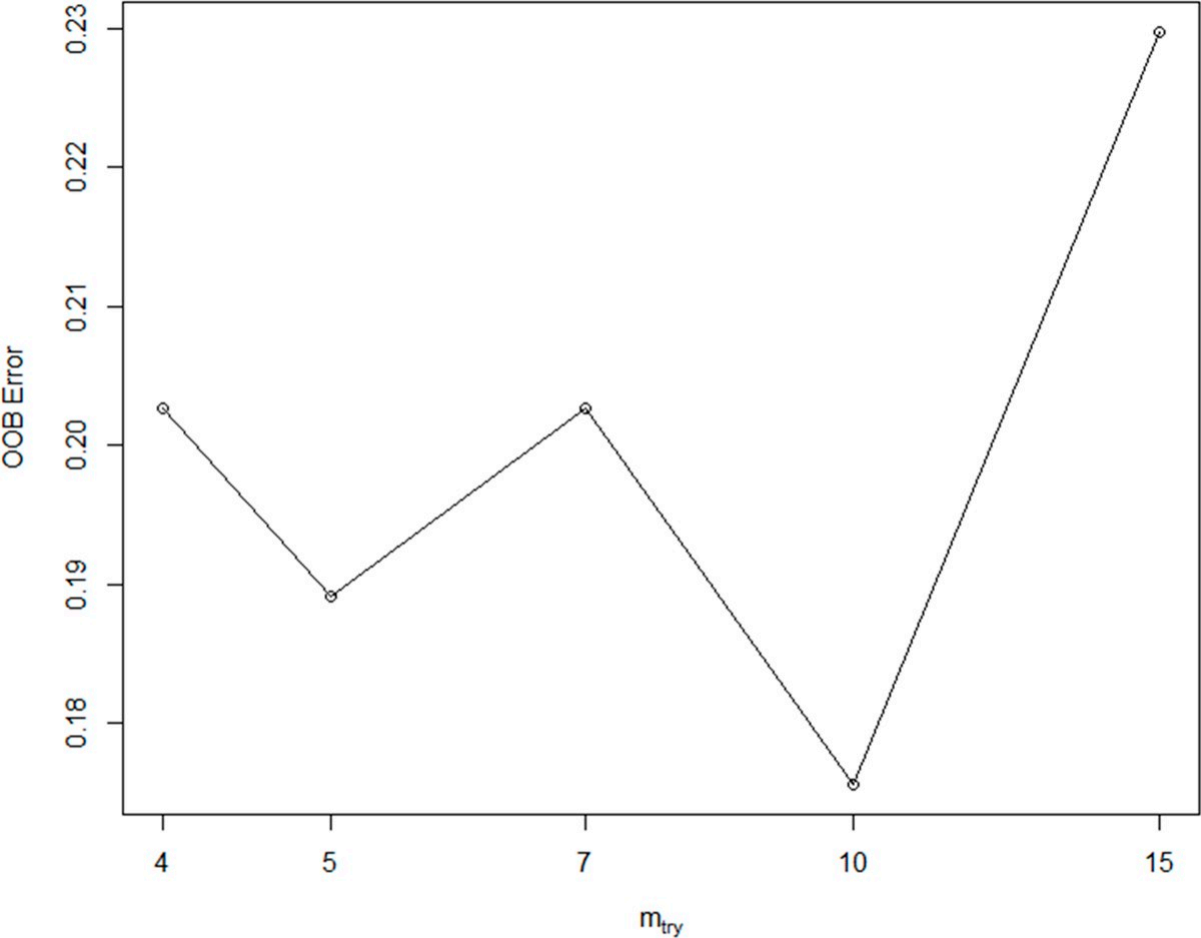
Relationship between Out-of-bag error and optimal mtry value for Random Forest.

**Fig 6:**
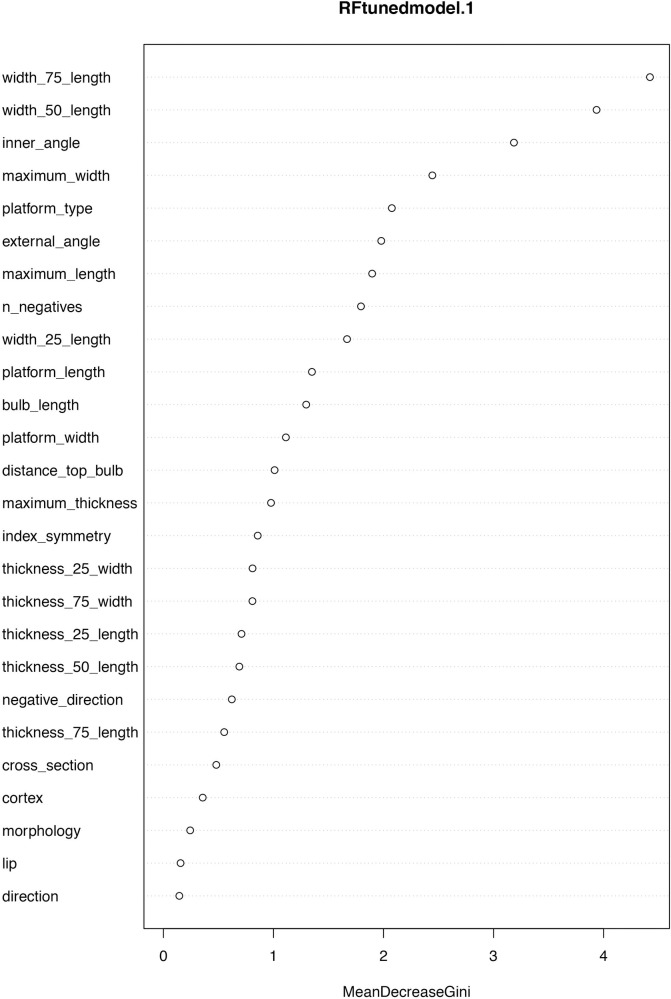
Importance of variables for the final Random Forest model, without including “technology classification” variable.

**Fig 7:**
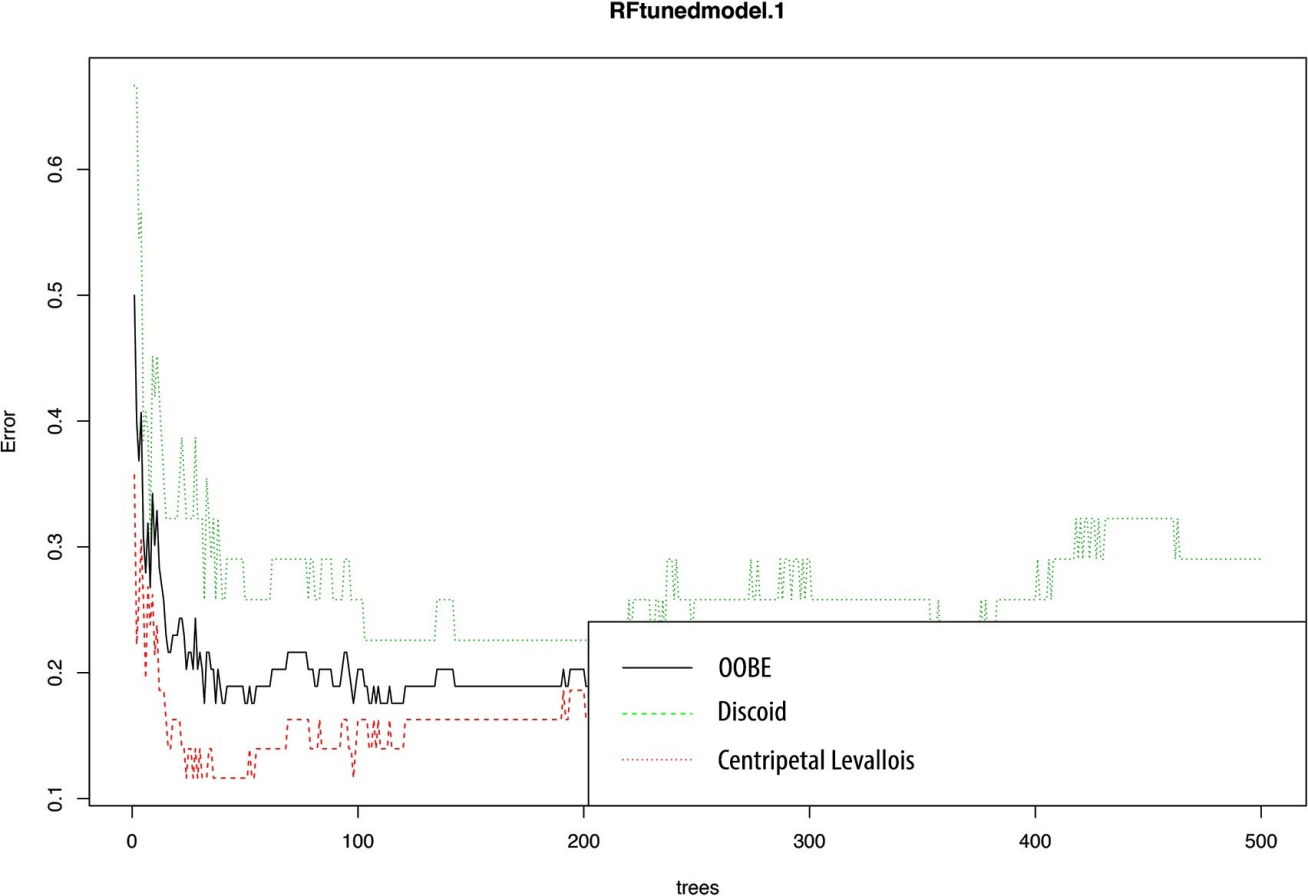
Error variation for both the two classes based on the number of trees.

Even though we successfully managed to improve the performance of most of the algorithms, we noticed the variable with highest impact in our classification was “technology classification” (Fig 4); because this feature depends entirely on the researcher, we implemented the algorithms for a third time, using as parameters the ones obtained from the hyperparameter optimization, but without including this variable. Considering this, most of the algorithms decreased their performance, but keeping, however, high accuracies: 80% for RF, MDA and DTC5.0, 76% for SVM and PLS and 73% for KNN and NB (Table 5). Thus, we selected RF as our final model since it was one of the most accurate and allows to search for the optimal number of variables. In this case, mtry = 10 was the best option since it showed an OOBError of 0.1756757. Therefore, our final classification achieved 80% accuracy considering only 10 variables (Figs 5–7).

**Table 5:** Details on the performance of the seven Machine Learning algorithms compared after hyperparameter optimization and without “technology classification” variable.

Algorithm	Accuracy	Kappa	95%CI	Sensitivity	Specificity
Support vector machine	77%	0.53	0.58–0.90	0.72	0.83
K-nearest neighbor	73%	0.46	0.54–0.88	0.72	0.75
Random Forest	80%	0.59	0.61–0.92	0.78	0.83
Mixture discriminant analysis	80%	0.59	0.61–0.92	0.78	0.83
Naive Bayes	70%	0.40	0.51–0.85	0.67	0.75
Partial least squares discriminant analysis	76%	0.53	0.58–0.90	0.72	0.83
Decision tree with C5.0 algorithm	80%	0.58	0.61–0.92	0.83	0.75

As we can see, the sequence of experiments we have carried out demonstrates that the null hypothesis of Discoid and Centripetal Recurrent Levallois methods as almost indistinguishable must be rejected, since all of the machine learning methods used can stablish differences with a degree of accuracy over 73% of the testing set.

## Discussion

Identifying the different knapping methods is an essential piece of information when it comes to understanding the economic behavior of the groups during the Palaeolithic. We are aware that, formally, any given type of blank can be obtained using very different methods. Therefore, it is essential to study lithic assemblages from a global point of view [1, 4, 61].

As we have already mentioned above, there is a real problem when identifying two lithic productions: Discoid and Centripetal Levallois, especially when both occur in the same assemblage. The techno-economic implications are different if one or the other method is used in relation to the débitage and raw material economy [41, 62] or even to the techno-economic traditions of the Middle Palaeolithic/MSA groups [63–65].

The study that we present here complements traditional technological studies. It allows discerning, with 80% of accuracy, both productions considering only ten parameters, some of them usually analyzed in all lithic collection studies.

Of the parameters selected (Fig 6), some typometric criteria seem to be particularly relevant. The importance of the width at 50% and 75% stands out, which shows that the proportion of the flake is relevant when discriminating between methods. Also, although to a lesser extent, the parameters of width at 25%, maximum width, and maximum length are important. In addition, the 25% thickness is important too, a parameter that refers to the thickness in the proximal part of the flake, where the thickness is usually significant among discoid flakes [1, 66–68]. These results are in agreement with the importance given to the morphological aspect of the recurrent Levallois and Discoid blanks citing the regularity and thinner sections of the Levallois flakes [36, 67].

Also crucial is the inner angle of the flakes to differentiate both productions. This quality, associated with the direction of flaking, has already been reviewed by many authors [17, 18, 41] and some of them practically exclusively [15]. In our collection, Levallois flakes tend to have more open angles (in some cases exceeding 90°), while Discoid flakes tend to have more closed angles. The external angle of the flake, it is also significant, but its role is less decisive and here the trend in our collection is the opposite of that which occurs in the inner angle.

The type of platform, except for some authors [36] has not been a determining parameter, although it was significant for the Levallois productions [34, 37]. In our sample, faceted platforms are more common in Levallois débitage and dihedrals in Discoids. Plaints platforms had the same percentage in both methods. This parameter is the fifth in contribution when it comes to discerning between both productions, so although it does not remain exclusive, there does seem to be a certain trend.

A little analyzed parameter in technological studies on these methods is the number of negatives (scars) on the dorsal surface of flakes. However, it is the seventh parameter in importance to discern this type of productions. In our collection, the sample with the largest number of previous scars was the Centripetal Levallois sample, a fact that Perpère already marked as important in her studies [5].

In Fig 7 we can see how the algorithm needs a greater number of trees to stabilize the prediction, an aspect that it achieves much earlier in the Centripetal Levallois sample. Furthermore, the success rate is higher among the Levallois blanks (85%), while the Discoid sample is more complex to identify because it resembles the Levallois sample (75% accuracy).

The study of lithic technology is understood as an integrative process because it allows learning the technical knowledge and know-how that the stone tool makers applied during knapping thanks to concepts such as method, technique, processes, etc. [38, 69]. All of them are best understood through the *chaîne opératoire* concept. Therefore, the isolated artifact *per se* does not exist but is inserted in the production system [70]. In other words, processes are studied, not “states” [71].

In this sense, the analysis and methodology that we present, far from deriving in a "Technological Typology" or "technography", have proven to be a reliable tool that allows us to refine further the characterization and systems of lithic production of the two methods examined.

We are aware of the limitations of our study in terms of sample size, raw materials and knapper knowledge. However, we consider that we have taken a first step towards a new type of analysis that will allow us to better discern overlapping knapping methods in the Paleolithic lithic technology.

## Conclusions

We can draw the following conclusions from this study:

1. It is indeed possible to distinguish flakes from Discoid and Centripetal Levallois methods with a 80% of accuracy. To do this, our model proposes measuring only 10 parameters from each flake.
2. These parameters are, in order of importance: width 75%, width 50%, inner angle, maximum width, platform type, external angle, number of negatives (previous scars), maximum length, width 25% and thickness 25%.

This turns out to be an excellent supporting feature to the structural characteristics already defined for this type of methods [17, 42], always within the purpose of understanding lithic technology as a whole process and not as an attempt of technography based exclusively on morphology.

Although we are aware that the algorithm has been tested with highly controlled variables, it should be tested in the future with more variables such as different types of raw material, size and morphology of the stone nodules or the knapper skills. The aim of this study is to establish a framework that demonstrates that there are differences between both methods in controlled samples and, in this first approach, ML has proven to be very useful tool for the analysis of technological variables, as it has previously shown in other areas of Prehistory such as taphonomy.

## Supporting information

S1 Text Experiment supplementary data. (DOCX)

S2 Text Direction of negatives diagram. (DOCX)

S3 Text Technological classification. Definitions of the technological classification used in the analysis.
(DOCX)

S4 Text Accuracy, 95% CI, Kappa, specificity and sensitivity concepts. (DOCX)
