# Supplementary material for: Distinguishing Discoid and Centripetal Levallois methods through machine learning
Source: PLoS One. 2020 Dec 23;15(12):e0244288. doi: 10.1371/journal.pone.0244288 (PMC7757815; doi:10.1371/journal.pone.0244288)
Supplement: S1 Text — (DOCX) [file pone.0244288.s001.docx]

**Distinguising Discoid and Centripetal Levallois methods through Machine Learning**

Irene González-Molina, Blanca Jiménez-García, José-Manuel Maíllo-Fernández, Enrique Baquedano, Manuel Domínguez-Rodrigo.

**S1 Text. Experiment supplementary data.**

Univariate statistics on the typometry of the experimental collection (only exploitation phase flakes).

|  | **General** | **Discoid** | **Levallois** |
| --- | --- | --- | --- |
| **Min** | 13.00 | 13.00 | 22.00 |
| **Max** | 106.00 | 74.00 | 106.00 |
| **1^st^ Qu.** | 30.00 | 25.00 | 40.25 |
| **3^rd^ Qu.** | 57.00 | 49.00 | 65.00 |
| **Median** | 42.00 | 38.00 | 53.00 |
| **Mean** | 44.75 | 38.41 | 53.28 |
| **SD** | 17.88859 | 15.51724 | 17.38155 |

Bplot of six cores analyzed. CL = Centripetal Levallois, D = Discoid. Measures in centimeters.

**
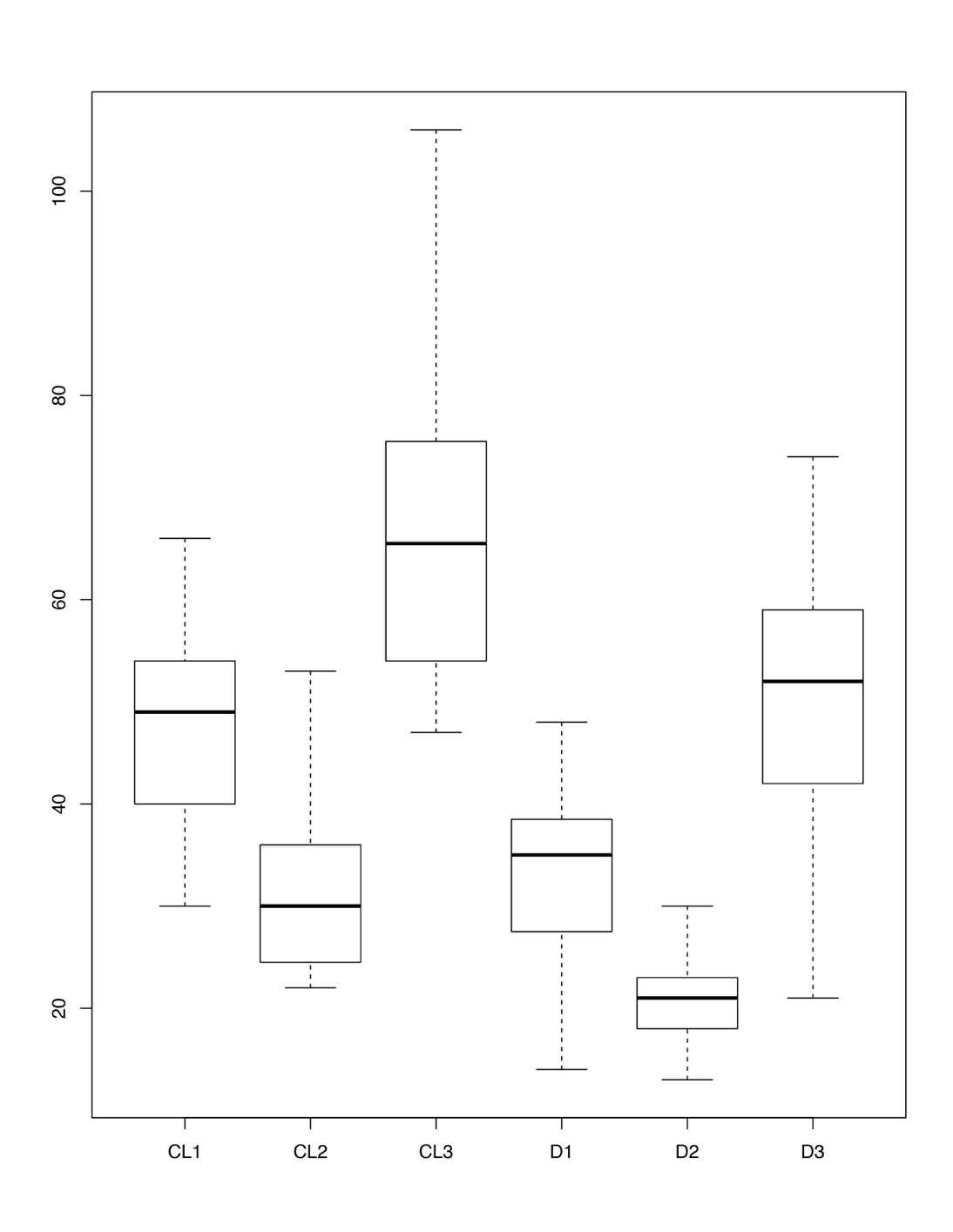
**

Example of Centripetal Levallois core and flakes (Experiment # 1): A) core; b) flakes.

**
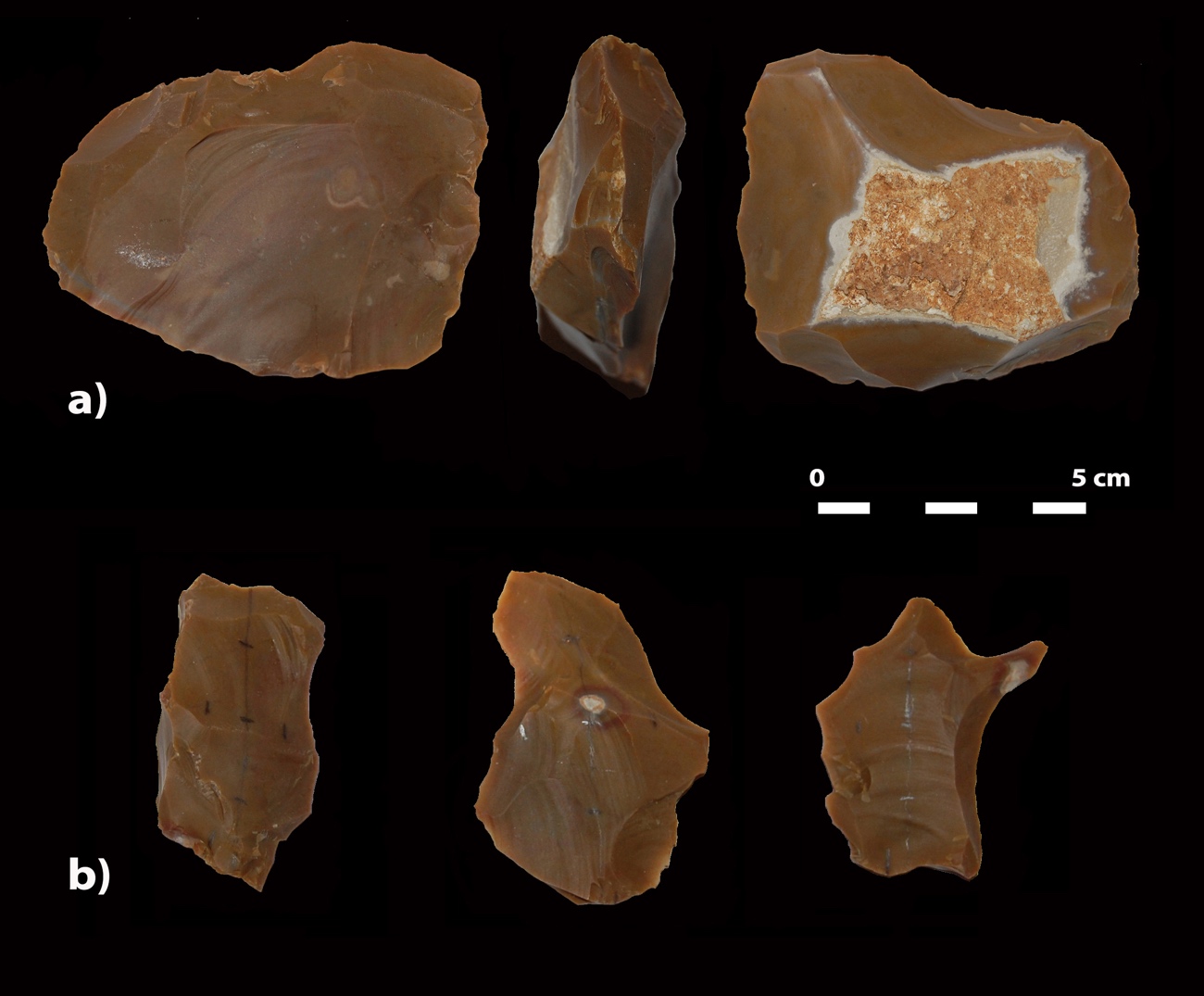
**

Example of Discoid core (experiment # 3): a) core; b) flakes.

**
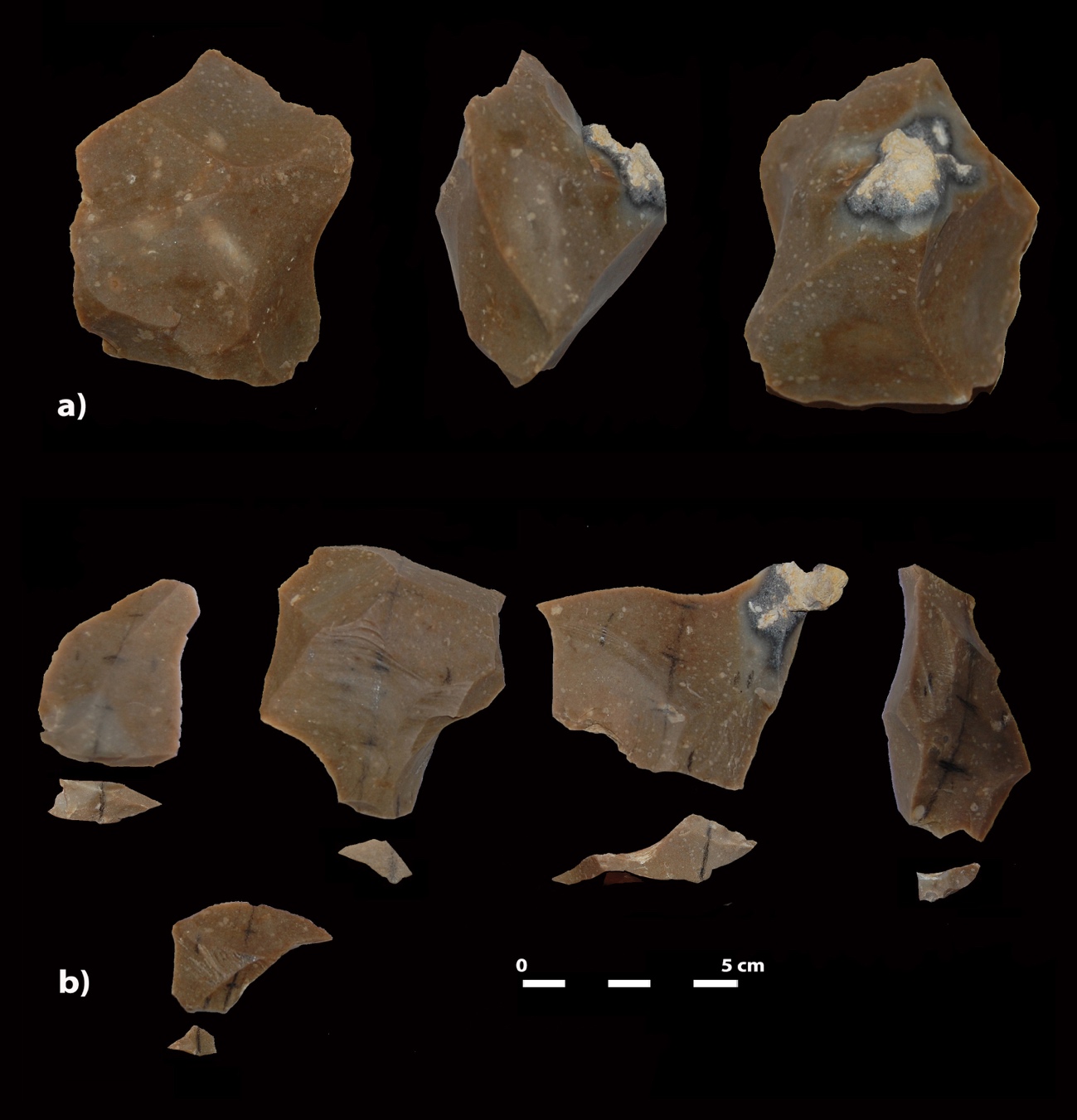
**
