# Supplementary material for: Distinguishing Discoid and Centripetal Levallois methods through machine learning
Source: PLoS One. 2020 Dec 23;15(12):e0244288. doi: 10.1371/journal.pone.0244288 (PMC7757815; doi:10.1371/journal.pone.0244288)

**Distinguising Discoid and Centripetal Levallois methods through Machine Learning**

Irene González-Molina, Blanca Jiménez-García, José-Manuel Maíllo-Fernández, Enrique Baquedano, Manuel Domínguez-Rodrigo.

**S2 Text. Direction of negatives diagram.**

Direction of negatives (previous scars). We distinguish between centripetal direction (1), distal (2), distal-lateral (3), proximal-lateral (4), proximal (5) and bidirectional (6).


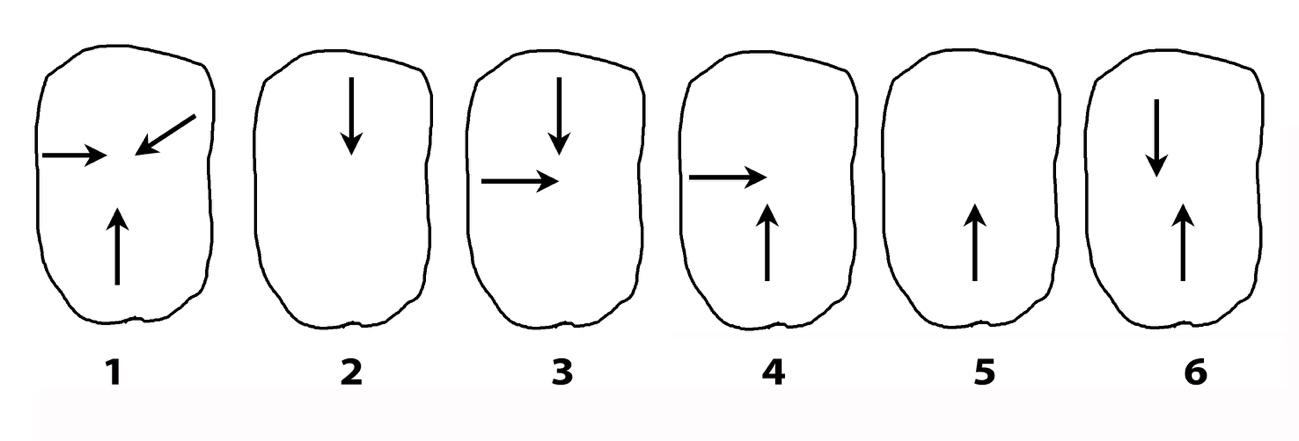

Supplement: S2 Text — (DOCX) [file pone.0244288.s002.docx]
