# Supplementary material for: Distinguishing Discoid and Centripetal Levallois methods through machine learning
Source: PLoS One. 2020 Dec 23;15(12):e0244288. doi: 10.1371/journal.pone.0244288 (PMC7757815; doi:10.1371/journal.pone.0244288)
Supplement: S3 Text — Definitions of the technological classification used in the analysis. (DOCX) [file pone.0244288.s003.docx]

**Distinguising Discoid and Centripetal Levallois methods through Machine Learning**

Irene González-Molina, Blanca Jiménez-García, José-Manuel Maíllo-Fernández, Enrique Baquedano, Manuel Domínguez-Rodrigo.

**S3 Text. Technological classification.**

Technological classification used in the analysis. Modified after [1-3].

1. *Débordant* flake. Flakes removed along the lateral edge of the debitage surface from discoidal or Levallois cores in order to create the lateral convexity of the core.

2. Cordal Flake. As *débordant* flake but the flake removed is tangential to core’s longitudinal axis. In both types of methods, it maintains convexity. The morphological and technological axes do not coincide.

3. Pseudolevallois Point. As cordal flake but the flake is pointed. The morphological and technological axes do not coincide.

4. Centripetal flake. Centripetal direction flakes are discoid blanks. Usually they are square or rectangular (wider than long). Their longitudinal section is triangular.

5. Centripetal Levallois Flake. The blanks of Centripetal Levallois would be varied both morphologically and metrically. The panoply varies from symmetric blanks in the first series of debitage (similar to the preferential Levallois flakes), to atypical, centripetal, *débordant* Levallois flakes (with cortical back or not) and pseudolevallois points.

1. Boëda E. Le débitage discoide et le débitage levallois récurrent centripéte. Bulletin de la Société Préhistorique Francaise. 1993; 90(6): 392–404.
2. Maíllo-Fernández JM, Solano-Megías I, Mabulla A, Arriaza MC, Bower JFR. Lithic Technology at Loiyangalani, a Late Middle Stone Age Site in the Serengeti, Tanzania. African Archaeological Review. 2019; 36: 291-315.
3. Meignen L. The Mousterian Lithic Assemblage from Kebara Cave. In Meignen L, Bar-Yosef O, editors. Kebara Cave, Mt. Carmel, Israel. The Middle and Upper Paleolithic Archaeology, Part II. Harvard: American School of Prehistoric Research Bulletin 51; 2019. pp. 1-147.
