# Supplementary material for: Distinguishing Discoid and Centripetal Levallois methods through machine learning
Source: PLoS One. 2020 Dec 23;15(12):e0244288. doi: 10.1371/journal.pone.0244288 (PMC7757815; doi:10.1371/journal.pone.0244288)
Supplement: S4 Text — (DOCX) [file pone.0244288.s004.docx]

**Distinguising Discoid and Centripetal Levallois methods through Machine Learning**

Irene González-Molina, Blanca Jiménez-García, José-Manuel Maíllo-Fernández, Enrique Baquedano, Manuel Domínguez-Rodrigo.

**S4 Text. Accuracy, 95% CI, Kappa, specificity and sensitivity concepts**

Measures used to evaluate the performance of the algorithms, defined after [1-2]:

1. Accuracy. Number of correct assessments/ number of total assessments

2. Kappa value. Statistical parameter that considers the amount of accuracy generated by chance. It indicates a perfect correspondence between the model and the documented classes (Kappa =1) or an imperfect one (Kappa ≤ 1). Cohen’s Kappa value is more robust than accuracy when measuring since it does not quantify the level of agreement between different sets, but it shows the degree of similarity corrected by chance.

3. 95% CI. Interval in which is contained the parameter that is being estimated (in this case, with a 95% confidence)

4. Specificity. Number of true negatives / number of total negative assessments (true negatives + false positives)

5. Sensitivity. Number of true positives / number of total positive assessments (true positives + false negatives)

1. Domínguez Rodrigo M. Successful classification of experimental bone surface modifications (BSM) through machine learning algorithms: a solution to the controversial use of BSM in paleoanthropology? Archaeological and Anthropological Sciences. 2019; 11: 2711-2725.
2. Zhu W, Zeng N, Wang N. Sensitivity, Specificity, Accuracy, Associated Confidence Interval and ROC Analysis with Practical SAS® Implementations. Proceedings of the SAS Conference. 2010; 9.
